# Structure of the dihydrolipoamide succinyltransferase catalytic domain from *Escherichia coli* in a novel crystal form: a tale of a common protein crystallization contaminant

**DOI:** 10.1107/S2053230X19011488

**Published:** 2019-08-29

**Authors:** Babak Andi, Alexei S. Soares, Wuxian Shi, Martin R. Fuchs, Sean McSweeney, Qun Liu

**Affiliations:** a National Synchrotron Light Source II, Brookhaven National Laboratory, Upton, NY 11973-5000, USA; b Biology Department, Brookhaven National Laboratory, Upton, NY 11973-5000, USA

**Keywords:** dihydrolipoamide succinyltransferase, auxin, amidase, contaminant crystallization, protein crystallography, molecular replacement

## Abstract

The crystallization of amidase, the ultimate enzyme in the Trp-dependent auxin-biosynthesis pathway, from *Arabidopsis thaliana* was attempted using protein samples with at least 95% purity. Cube-shaped crystals that were assumed to be amidase crystals that belonged to space group *I*4 (unit-cell parameters *a* = *b* = 128.6, *c* = 249.7 Å) were obtained and diffracted to 3.0 Å resolution. Molecular replacement using structures from the PDB containing the amidase signature fold as search models was unsuccessful in yielding a convincing solution. Using the *Sequence-Independent Molecular replacement Based on Available Databases* (*SIMBAD*) program, it was discovered that the structure corresponded to dihydrolipoamide succinyltransferase from *Escherichia coli* (PDB entry 1c4t), which is considered to be a common crystallization contaminant protein. The structure was refined to an *R*_work_ of 23.0% and an *R*_free_ of 27.2% at 3.0 Å resolution. The structure was compared with others of the same protein deposited in the PDB. This is the first report of the structure of dihydrolipo­amide succinyltransferase isolated without an expression tag and in this novel crystal form.

## Abstract 2

Dihydrolipoamide succinyltransferase, a protein crystallization contaminant, was crystallized in a new crystal form in space group *I*4 and its structure was determined.

## 1. Introduction   

Most of the proteins used for X-ray crystallography are expressed in *Escherichia coli* with a polyhistidine fusion peptide and are purified using immobilized metal-ion affinity chromatography (IMAC) resins. The purity and homogeneity of the protein samples are critical in defining the outcome of a crystallization trial. Several reports in the literature have described the unfortunate crystallization of contaminating proteins at very low concentrations (Bolanos-Garcia & Davies, 2006 ▸; Cámara-Artigas *et al.*, 2006 ▸; van Eerde *et al.*, 2006 ▸; Veesler *et al.*, 2008 ▸; Keegan *et al.*, 2016 ▸; Niedzialkowska *et al.*, 2016 ▸). Acriflavin resistance protein B (AcrB) from *E. coli* was reported to be a major problem in membrane-protein crystallization trials owing to its high affinity for IMAC resins and its high degree of crystallizability (Veesler *et al.*, 2008 ▸). Bacterioferritin from *E. coli* is another reported crystallization contaminant protein. Red crystals of bacterioferritin were observed during the crystallization of *Sulfolobus acido­caldarius* 2-keto-3-deoxygluconate aldolase (van Eerde *et al.*, 2006 ▸). Cámara-Artigas *et al.* (2006 ▸) reported that when they tried to crystallize ferrodoxin-dependent glutamate synthase from spinach leaves, they observed two crystal forms of glyceraldehyde-3-phosphate dehydrogenase (GADPH). The structure determination of a contaminant periplasmic phosphate-binding (PBP) protein from *Stenotrophomonas maltophilia* inspired the development of the program *SIMBAD* (*Sequence-Independent Molecular replacement Based on Available Databases*; Keegan *et al.*, 2016 ▸). *ContaMiner* (a web server) and ContaBase (a contaminant database) allow the rapid screening of crystallographic data based on molecular replacement against 62 currently known contaminants (Hungler *et al.*, 2016 ▸).

In our attempts to crystallize amidase (EC 3.5.1.4) from *Arabidopsis thaliana* (UniProt accession No. Q9FR37; Pollmann *et al.*, 2003 ▸; Neu *et al.*, 2007 ▸), we serendipitously crystallized the catalytic domain of *E. coli* dihydrolipoamide succinyltransferase (EC 2.3.1.61), which belongs to the family of α-keto acid dehydrogenase complexes, in a novel crystal form and without an expression tag.

Three extremely large enzyme complexes in the family of α-keto acid dehydrogenase multi-enzymes have been described (Reed & Hackert, 1990 ▸; Perham, 1991 ▸): (i) α-ketoglutarate dehydrogenase complexes (KGDCs), (ii) pyruvate dehydro­genase complexes (PDCs) and (iii) branched-chain α-keto acid dehydrogenase complexes (BCKDCs). Each complex contains multiple copies of three enzymes. The first enzyme (E1) is a decarboxylase–dehydrogenase with high substrate specificity. The second enzyme (E2) is a unique dihydro­lipo­amide succinyltransferase. The third enzyme (E3) is a common dihydrolipoamide dehydrogenase (Knapp *et al.*, 2000 ▸). To form an active complex, multiple copies of the E1 and E3 subunits attach to a core complex made of only E2 sub­units. Two physiologically relevant polyhedral arrangements of E2 subunits have been observed to date. One arrangement is made of 24 E2 subunits with an octahedral symmetry and the other is made of 60 E2 subunits with an icosahedral symmetry; both arrangements utilize trimers as building blocks (Mattevi *et al.*, 1992 ▸; Knapp *et al.*, 1998 ▸).

The E2 subunit contains three domains. The N-terminal domain is a lipoyl-binding domain which may be composed of one, two or even three repeating lipoyl-binding domain units followed by an E1/E3-binding domain. The C-terminal domain is a catalytic domain. The E2 enzyme is highly modular and all of the domains are assembled together with very flexible linker segments, which makes the crystallization of intact E2 subunits extremely difficult (Reed & Hackert, 1990 ▸; Perham, 1991 ▸). In the case of the E2 subunit of the α-ketoglutarate dehydrogenase complex (KGDC) from *E. coli*, NMR structures of the lipoyl domain (PDB entry 1pmr; Ricaud *et al.*, 1996 ▸) and of the E3-binding domain (PDB entry 1bbl; Robien *et al.*, 1992 ▸), and crystal structures of the catalytic domain in a trimeric form (PDB entry 1c4t; Knapp *et al.*, 2000 ▸), in a physiologically relevant 24-mer core complex form (PDB entry 1e2o; Knapp *et al.*, 1998 ▸) and in an improved 24-mer form (PDB entry 1scz; N. Schormann, J. Symersky, M. Carson, M. Luo, J. Tsao, D. Johnson, W.-Y. Huang, P. Pruett, G. Lin, S. Li, S. Qiu, A. Arabashi, B. Bunzel, D. Luo, L. Nagy, R. Gray, C.-H. Luan, Z. Zhang, S. Lu & L. DeLucas, unpublished work) have been reported.

In this communication, we report the crystal structure of the dihydrolipoamide succinyltransferase catalytic domain from *E. coli* (*Ec*DSCD; PDB entry 6pbr) without an expression tag and compare this structure with the known PDB entries for the same protein. Crystal-packing analysis shows an arrangement of a physiologically relevant 24-mer core complex in this structure, which provides additional insights into the organization of the E2 subunit of the *E. coli* KGDC.

## 2. Materials and methods   

### 2.1. Protein expression and purification   

The amidase from *A. thaliana* (UniProt accession No. Q9FR37) was expressed in *E. coli* BL21 (DE3) pLysS strain grown in Terrific Broth (TB) medium using a pNYCOMPSC-23 expression plasmid. The plasmid was sequenced to confirm the correctness of the target gene. For expression, TB medium (2 × 1 l) containing 34 µg ml^−1^ chloramphenicol and 100 µg ml^−1^ carbenicillin was freshly inoculated with 100 ml overnight culture and incubated at 310 K with constant shaking until an OD_600_ of ∼1.1 was reached. Protein expression was induced using 1.0 m*M* isopropyl β-d-1-thiogalactopyranoside (IPTG) at 310 K for ∼4 h with constant shaking. The cells were harvested by centrifugation at 6000 rev min^−1^ for 5 min with a Sorvall GS-3 rotor at 277 K. The pellets were suspended in 50 ml lysis buffer (50 m*M* Tris pH 8.0, 300 m*M* NaCl), aliquoted into 2 × 25 ml Falcon tubes and immediately frozen until further analysis. A summary of protein production is shown in Table 1 ▸.

**Figure 1:**
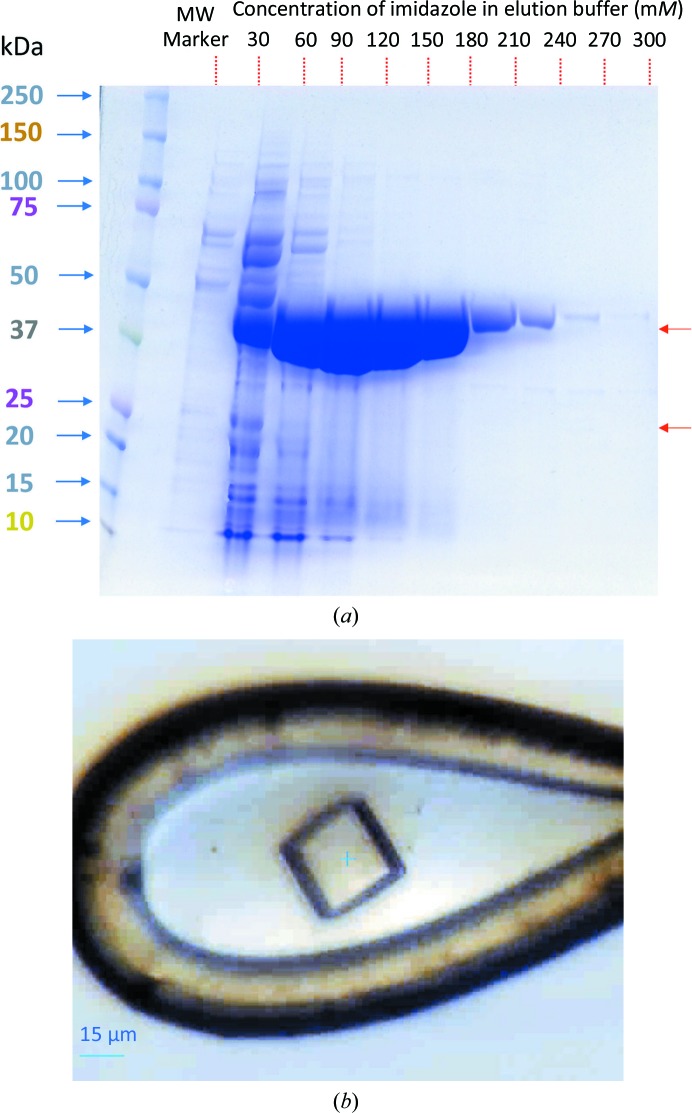
Protein production and crystallization. (*a*) SDS–PAGE analysis of the amidase elution fractions showing a trace amount of a contaminant which possibly contains *Ec*DSCD. Fractions showing a purity of greater than 95% (120–300 m*M* imidazole) were pooled and further concentrated to 254 mg ml^−1^ as measured by the absorbance at 280 nm. The molecular weight of the His-tagged amidase is 47.3 kDa. The concentration of *Ec*DSCD (the crystallized impurity) is roughly estimated as <0.1–0.2 mg ml^−1^ in a 10× concentrated amidase solution based on a band with a molecular weight of 26 kDa (we speculate that it is the band visible in the 30 m*M* imidazole elution lane). Red arrows show the approximate locations of the uncleaved and cleaved transferase based on molecular weights of 44 and 26 kDa, respectively. The image was analysed using *ImageJ* v.1.51j8 (https://imagej.nih.gov/ij/) to estimate the concentrations of total impurities and of *Ec*DSCD. (*b*) A crystal of *Ec*DSCD as mounted on a cryo-loop at the beamline. The crystal is roughly 35 × 35 × 30 µm in size.

The cells were lysed by sonication (Fisher Scientific 550 sonic dismembrator) with six bursts (10 s duration per burst with 30–40 s intervals) on ice. The sample was then centrifuged (18 000 rev min^−1^, 20 min, 277 K, Sorvall SS-34 rotor). The supernatant was directly applied onto Ni–NTA resin equilibrated with lysis buffer on a gravity column. The resin was washed with lysis buffer (approximately ten times the resin volume) before elution. A stepwise gradient of 30–300 m*M* imidazole in lysis buffer was used for elution. Amidase-containing fractions with a purity of >95% based on SDS–PAGE analysis [Fig. 1 ▸(*a*)] were then pooled together and concentrated to 254 mg ml^−1^ using an Amicon ultrafiltration unit (Millipore filter with 10 kDa molecular-weight cutoff) and a centrifugal filter device (Amicon Ultracel with 10 kDa molecular-weight cutoff).

### 2.2. Crystallization   

A summary of the crystallization conditions is shown in Table 2 ▸. Small cube-shaped crystals appeared after 40 days and reached their maximum dimensions in about three months. For cryocooling, a cryosolution consisting of 25 m*M* Tris pH 8.0, 1.3 *M* NaCl, 25% glycerol was used and the crystals were immediately flash-cooled in liquid nitrogen.

### 2.3. Data collection and processing   

Data were indexed, integrated and scaled with *FastDP* (based on *XDS*; Kabsch, 2010*a* ▸,*b* ▸; Grosse-Kunstleve *et al.*, 2002 ▸; Winter & McAuley, 2011 ▸; Winn *et al.*, 2011 ▸). The Matthews coefficient (*V*_M_) was calculated as 3.33 Å^3^ Da^−1^, corresponding to six monomers (two trimers; one trimer is located in each corner or apex of the cubic 24-mer particle) per asymmetric unit with an estimated solvent content of 63%. A summary of the data-collection statistics is shown in Table 3 ▸.

### 2.4. Structure solution and refinement   

We used *Phaser* (McCoy *et al.*, 2007 ▸) as implemented in *CCP*4 (Winn *et al.*, 2011 ▸) for molecular replacement. Full-length and truncated structures in the PDB containing the amidase signature fold, including PDB entries 2dc0 (LLG = 6.0, TFZ = 3.8; RIKEN Structural Genomics/Proteomics Initiative, unpublished work), 4wj3 (LLG = 26.0, TFZ = 3.1; Suzuki *et al.*, 2015 ▸), 6c62 (LLG = 33.0, TFZ = 4.5; Esquirol *et al.*, 2018 ▸) and 3al0 (LLG = 27.0, TFZ = 3.4; Ito & Yokoyama, 2010 ▸), were used as molecular-replacement search models without any convincing solutions. The maximum log-likelihood gradient (LLG) and translation-function *Z*-score (TFZ) are as reported by *Phaser*. After multiple unsuccessful attempts, we decided to use the *SIMBAD* program (Simpkin *et al.*, 2018 ▸), which uses *MOLREP* (Vagin & Teplyakov, 2010 ▸) and *AMoRe* (Navaza, 1994 ▸) as its underlying programs, to search for a solution. *SIMBAD* did not find a positive hit from its unit-cell parameter search as the structure has no lattice match in the existing PDB. However, the contaminant search option in the program identified PDB entry 1c4t (Knapp *et al.*, 2000 ▸ ) as a possible solution (*Z*-score = 9.7, *R*_free_ = 0.31). We then solved the structure by molecular replacement using a monomer from PDB entry 1c4t (LLG = 307, TFZ = 16.4) as a search model. Model building and refinement were carried out using *Coot* (Emsley *et al.*, 2010 ▸) and *REFMAC*5 (Murshudov *et al.*, 2011 ▸). The structure was validated using *PROCHECK* (Laskowski *et al.*, 1993 ▸). A summary of refinement statistics and model validation values are shown in Table 4 ▸. All molecular-graphics figures were created using *PyMOL* (v.2.3.0; Schrödinger).

## 3. Results and discussion   

### 3.1. *Ec*DSCD production   

Based on the SDS–PAGE, the amount of crystallized protein impurity was estimated to be less than 0.1–0.2 mg ml^−1^ in a 254 mg ml^−1^ solution of amidase [Fig. 1 ▸(*a*)], which is only ∼0.06% of the total protein content of the amidase solution. The molecular weight of the intact E2 monomer is 44 kDa; however, the molecular weight of the determined structure of the catalytic domain was 26 kDa (*i.e. Ec*DSCD). The structure lacks any observable electron density for the E3-binding domain, N-terminal lipoyl-binding domain or the associated linkers. It is likely that these domains are removed by endogenous proteases prior to or during the crystallization process. The crystallization of the 24-mer E2 component with a similar proteolytic cleavage has been reported previously, most likely owing to the same unwanted process at the same cleavage site (Knapp *et al.*, 1998 ▸, 2000 ▸). The reason for the capture of dihydrolipoamide succinyltransferase by affinity purification is not clear. Bolanos-Garcia & Davies (2006 ▸) suggested that the large oligomerization state of the dihydrolipoamide succinyltransferase component might provide a larger surface area for metal-affinity binding. The protein has some surface-accessible histidine residues which might bind to IMAC resin at pH 8.

The binding of dihydrolipoamide succinyltransferase to IMAC resins and its crystallization as a contaminant have been reported in previous studies (Bolanos-Garcia & Davies, 2006 ▸; Niedzialkowska *et al.*, 2016 ▸). To detect a contaminant protein, using a different affinity tag for purification, adding additional purification steps, a thorough search of the PDB using the *SIMBAD* molecular-replacement program and the use of other analytical methods such as protein sequencing and mass spectrometry (Veesler *et al.*, 2008 ▸) are the available options.

### 3.2. Crystallization   

A summary of the crystallization parameters of *Ec*DSCD in the PDB are shown in Table 5 ▸. We have crystallized this enzyme in a new condition and in a new crystal form. The new crystallization precipitant contained only NaCl, which differs from the previously reported crystallization conditions as shown in Table 5 ▸. At typical protein concentrations of 10–25 mg ml^−1^ no initial crystallization hit for the amidase was found. We thus used a tenfold higher concentratation of amidase, in which the concentration of *Ec*DSCD was high enough to produce crystals. It is also possible that the high concentration of amidase (254 mg ml^−1^) may play a role in the crystallization of *Ec*DSCD and therefore should be considered to be an integral part of the crystallization environment. Being able to concentrate the amidase enzyme to as high as 254 mg ml^−1^ without any precipitation or denaturation came as a surprise to us. We imagine that the high solubility of amidase might be related to its physiological significance under stress conditions. *Ec*DSCD crystallization was quite reproducible when the amidase concentration was higher than 200 mg ml^−1^; however, it took different lengths of time ranging from 40 days to nine months for the crystals to appear in the drop, which apparently relates to the concentration of the impurity and or the proteolytic activity necessary to produce a crystallizable fragment. The cube-shaped protein crystals [Fig. 1 ▸(*b*)] were extremely sensitive to radiation damage, perhaps owing to the high salt concentration (1.3 *M* NaCl) and the large 24-mer assembly.

### 3.3. Structure determination   

Initial phasing attempts using molecular replacement (MR) with the amidase signature fold as a search probe were unsuccessful in yielding a convincing solution. The use of computer-predicted models with a docked amidase sequence as search probes was also unsuccessful. Using *SIMBAD*, a contaminating protein with PDB entry 1c4t was identified as a candidate model for molecular replacement. Even though *Ec*DSCD is a well known highly crystallizable contaminant, its structure had never been reported in an *I*4 crystal form and *SIMBAD* did not find a hit when only searching the PDB unit-cell parameter database.

Using a monomer from PDB entry 1c4t, *Phaser* located six molecules in the asymmetric unit of the crystal as two trimers [Supplementary Fig. S1(*a*)]. The two trimers of the asymmetric unit have slightly different overall conformations (r.m.s.d. on C^α^ atoms of 0.4 Å; not shown). Because of this conformational change, data in the *I*4 crystal form could not be processed in *I*422 and the packing is different from the previously reported *F*432 crystal form.

Samples of the electron-density map around the active site of monomer *A* and along the threefold NCS axis in the *I*4 crystal form are shown in Supplementary Figs. S1(*b*) and S1(*c*). His375, Asp379 and Glu382 are part of the region 4 active site and are highly conserved across species (Knapp *et al.*, 2000 ▸).

Solving the structure of *Ec*DSCD in an *I*4 crystal form using *SIMBAD* emphasizes the robustness of this MR program, making it easier to solve difficult cases (Simpkin *et al.*, 2018 ▸).

### 3.4. Active-site structure   

**Figure 2:**
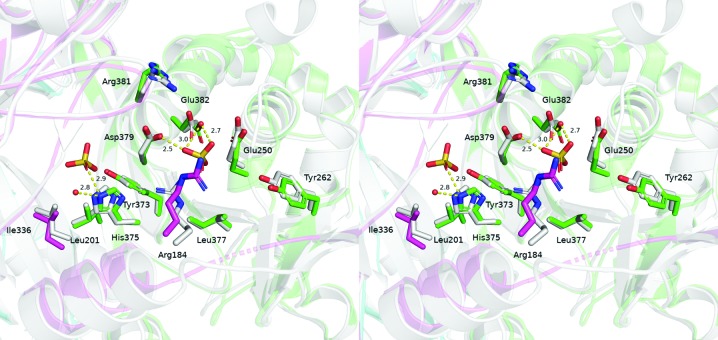
A stereo figure of the superposition of the region 4 active site of *Ec*DSCD in *P*3_1_21 (sulfate-bound; PDB entry 1c4t) and *I*4 (PDB entry 6pbr; this work) crystal forms. Chains *A*, *B* and *C* from PDB entry 1c4t are shown in green, cyan and purple, respectively. All of the chains from this work are shown in light grey. Sulfate molecules bind to three residues of the active site: His375, Asp379 and Glu382.

Superimposition of the active site of the enzyme is shown in Fig. 2 ▸. The sulfate-bound structure in the *P*3_1_21 crystal form is shown in green and purple, and the sulfate molecules bind to the three residues of the active site (Knapp *et al.*, 2000 ▸). The structure of the enzyme in the *I*4 crystal form (PDB entry 6pbr, this work) is shown in light grey, revealing some slight variabilities in side-chain conformation. It has been suggested that His375 initiates the first step of catalysis by deprotonating the thiol group of coenzyme A (CoA), which then attacks the carbonyl C atom of the succinylated dihydrolipoyl moiety. Breakdown of the intermediate will produce succinylated CoA and a protonated dihydrolipoyl group (Knapp *et al.*, 2000 ▸). It has also been suggested that Asp379 forms a substrate-dependent salt bridge with either His375 or Arg381, facilitating protonation/deprotonation events (Knapp *et al.*, 2000 ▸). A complex structure with the substrate CoA is needed to further the analysis of the proposed chemical mechanism.

### 3.5. Comparative structural analysis   

**Figure 3:**
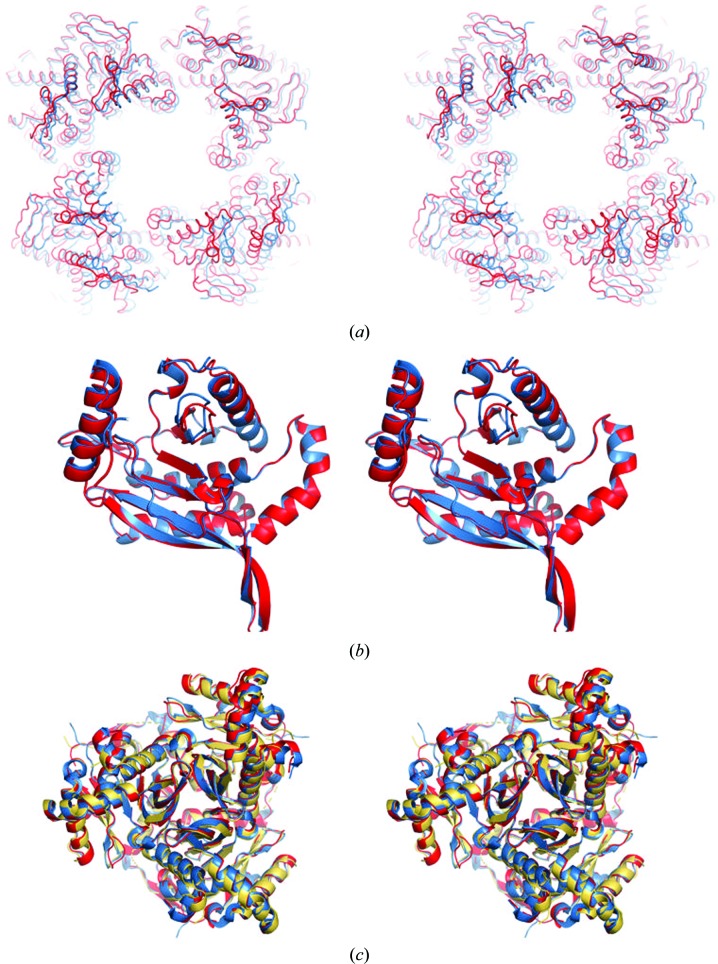
Structure and assembly of *Ec*DSCD. (*a*) Superposition (stereo figure with depth cue) of the 24-mer biological assemblies of *Ec*DSCD in the *F*432 (sky blue; PDB entry 1scz) and *I*4 (red; this work) crystal forms with respect to monomer *A*. For clarity, only the top 12-mer is shown as ribbons. The physiological significance of the observed structural changes is currently unknown, and these structural differences may arise from a different molecular packing under our crystallization conditions. (*b*) Stereoview of the superimposition of monomer *A* in the *F*432 (blue) and *I*4 (red) crystal forms. Significant structural changes are present in some loops; however, the overall structures are very similar (r.m.s.d. of 0.3 Å). The structural differences seen in the 24-mer superpositions arise from the differences in these loop-mediated intermolecular interactions, as well as the intermolecular rotational and translational changes. (*c*) Stereoview of the superposition of the trimers in *P*3_1_21 (PDB entry 1c4t, yellow–orange), *F*432 (PDB entry 1scz, sky blue) and *I*4 (PDB entry 6pbr, red) crystal forms. Some conformational changes exist in helices and loop areas.

The superimposition of a 24-mer biological assembly of *Ec*DSCD (PDB entry 1scz) in the *F*432 crystal form with the corresponding assembly in the *I*4 crystal form (PDB entry 6pbr, this work) is shown in Fig. 3 ▸(*a*). While significant structural differences exist at the level of biological assemblies [Fig. 3 ▸(*a*)], the monomeric forms [Fig. 3 ▸(*b*)] superimpose well, with an r.m.s.d. of 0.3 Å for aligned C^α^ atoms. A trimer–trimer superposition in different crystal forms reveals some slight conformational changes [Fig. 3 ▸(*c*)]. Even in the *I*4 crystal form, the two trimers of the asymmetric unit adopt slightly different conformations, in which one trimer rotationally expands away around the threefold NCS axis of the trimer in comparison to the other trimer. Consequently, reconstruction of the 24-mer biological assembly using the asymmetric unit trimers and the fourfold crystallographic symmetry leads to a 24-mer that is slightly stretched outwards (skewed) compared with the 24-mer in the *F*432 form.

Our comparative structural analysis shows slight differences between the monomers in the different crystal forms; however, this translates into more significant changes in the bio-assemblies in the three known crystal forms of this protein. The physiological significance of these conformational changes is not clear, and may be relevant to the entire complex assembly, including binding of the E1 and E3 components. Based on the modelling of a coenzyme A (CoA) molecule into the active site, we also speculate that the structure reported in this work (PDB entry 6pbr) might be compatible with substrate binding; however, the structure of substrate-bound *Ec*DSCD awaits further studies.

### 3.6. Molecular-packing analysis   

**Figure 4:**
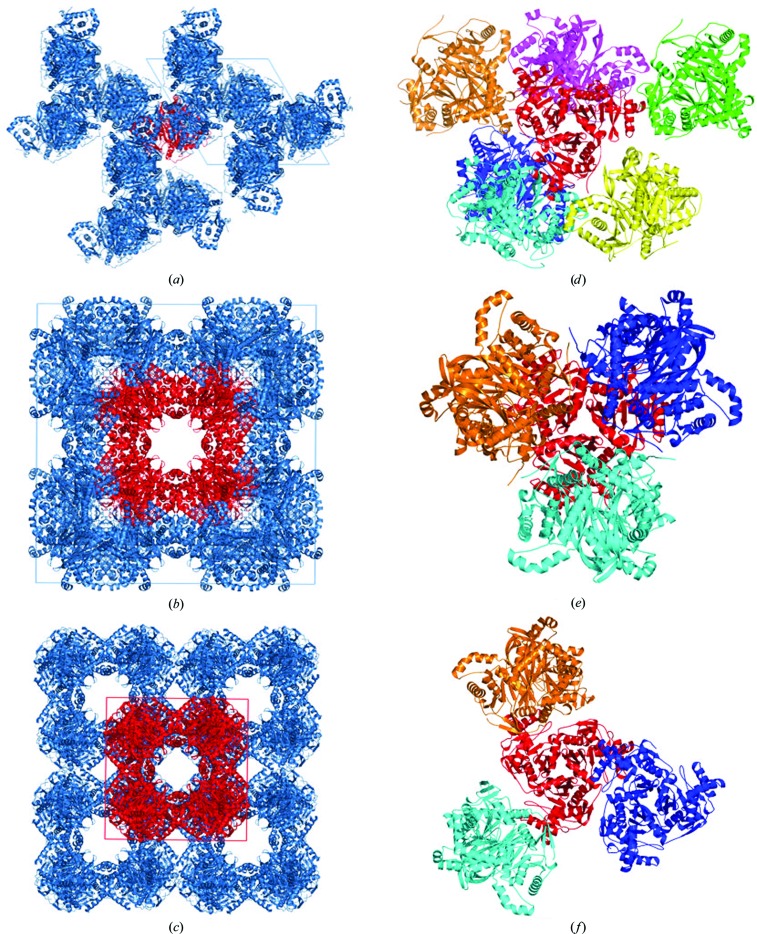
Molecular packing of *Ec*DSCD in three different crystal forms. (*a*) In space group *P*3_1_21 (PDB entry 1c4t), the trimer (in red) cannot form the 24-mer biological assembly. The dimensions of the unit cell (blue lines) shown in the plane are *a* = *b* = 112.18 Å. (*b*) In space group *F*432 (PDB entry 1scz), the packing forms a 24-mer biological assembly (in red). The dimensions of the unit cell (blue lines) shown in the plane are *a* = *b* = 220.58 Å. (*c*) In space group *I*4 (PDB entry 6pbr, this work), the packing shows a 24-mer biological assembly (in red). The dimensions of the unit cell (red lines) shown in the plane are *a* = *b* = 128.60 Å. The building block for all of the crystal forms of *Ec*DSCD is a trimer [shown in red in the *P*3_1_21 (*d*), *F*432 (*e*) and *I*4 (*f*) crystal forms]. Intermolecular interactions between the trimers are unique in the different crystal forms, as shown, while the monomer/trimer itself shows a minimal conformational change between crystal forms [Figs. 3 ▸(*b*) and 3 ▸(*c*)]. One trimer (in red) is depicted in the same orientation in all three structures for an easier comparative view.

A comparison of the molecular packing of the three known *Ec*DSCD structures in different crystal forms is shown in Figs. 4 ▸(*a*)–4 ▸(*c*). Trimers of the *P*3_1_21 crystal form with one trimer per asymmetric unit cannot form the 24-mer biological assembly owing to a His tag in their C-terminus, which leads to a different trimer–trimer interaction, as shown in Fig. 4 ▸(*d*), which depicts all of the interactions between trimers. For improved clarity, the trimers shown in Figs. 4 ▸(*e*) and 4 ▸(*f*) each originate from a different 24-mer biological assembly. The assembly in the *F*432 crystal form [Figs. 4 ▸(*b*) and 4 ▸(*e*)] contains one monomer in the asymmetric unit. The assembly in the *I*4 crystal form [Figs. 4 ▸(*c*) and 4 ▸(*f*)] contains six monomers (two trimers) in the asymmetric unit.

The expression vector for the trimeric enzyme crystallized in the *P*3_1_21 crystal form was constructed to include residues 93–404, lacking the N-terminal lipoyl-binding domain, with a molecular weight of 37 kDa (Knapp *et al.*, 2000 ▸). The crystallized enzyme, however, consists of residues 172–404, lacking additional N-terminal residues that belong to the E3-binding domain. The reason is the apparent release of the E3-binding domain by an endogenous protease prior to or during crystallization. The same construct with an N-terminal His tag can form a 24-mer biological assembly, as deduced from size-exclusion gel chromatography (Knapp *et al.*, 2000 ▸). A similar construct (residues 93–404) purified using a GST (glutathione *S*-transferase) tag has been crystallized and diffracted to 3.0 Å resolution in an *F*432 crystal form (PDB entry 1e2o), which forms a cubic core 24-mer biological assembly. This crystal structure also lacks the E3-binding domain and only residues 172–404 are visible in the structure (Knapp *et al.*, 1998 ▸). An improved structural model for *Ec*DSCD with 2.2 Å resolution is also available as PDB entry 1scz, which forms a 24-mer assembly containing residues 172–404. In the *I*4 crystal form (PDB entry 6pbr, this work) we observed a 24-mer biological assembly that also contains residues 172–404, suggesting that similar proteolytic digestion took place prior to or during crystallization.

## Supplementary Material

PDB reference: dihydrolipoamide succinyltransferase, 6pbr

Supplementary Figure S1. DOI: 10.1107/S2053230X19011488/rf5022sup1.pdf

## Figures and Tables

**Table 1:** Macromolecule-production information

Macromolecule	Amidase	Dihydrolipoamide succinyltransferase
Source organism	*A. thaliana*	*E. coli*
DNA source	*A. thaliana*	*E. coli*
Forward primer	TTAAGAAGGAGATATACTATGGCGACGAATAACGACTTCGGG	N/A
Reverse primer	TGAAAATAGAGGTTTTCGGCAATGAACGCTGCCAAACTGTCGAC†	N/A
Cloning vector	N/A	N/A
Expression vector	pNYCOMPSC-23	N/A
Expression host	*E. coli*	*E. coli*
Amino-acid sequence	UniProt Q9FR37	UniProt P0AFG6 (residues 173–405)

† C-terminal 10×His tag.

**Table 2:** Crystallization of dihydrolipoamide succinyltransferase

Method	Hanging-drop vapour diffusion
Plate type	VDX plate
Temperature (K)	298
Protein concentration	∼0.1–0.2 mg ml^−1^ (in a 254 mg ml^−1^ solution of amidase)
Buffer composition of protein solution	50 m*M* Tris pH 8.0, 300 m*M* NaCl, 254 mg ml^−1^ amidase
Composition of reservoir solution	2.6 *M* NaCl
Volume and ratio of drop	4 µl, 1:1 ratio
Volume of reservoir (ml)	1

**Table 3:** Data collection and processing Values in parentheses are for the outer shell.

Diffraction source	17-ID-1 AMX beamline, NSLS-II
Wavelength (Å)	0.92
Beam size (µm)	5 × 7
Transmission (%)	10
Temperature (K)	100
Detector	EIGER 9M
Crystal-to-detector distance (mm)	300
Rotation range per image (°)	0.2
Total rotation range (°)	80
Exposure time per image (s)	0.01
Space group	*I*4
*a*, *b*, *c* (Å)	128.60, 128.60, 249.73
α, β, γ (°)	90, 90, 90
Mosaicity (°)	0.12–0.21
Resolution range (Å)	47.32–3.00 (3.16–3.00)
Total No. of reflections	125185 (18229)
No. of unique reflections	33570 (5050)
Completeness (%)	82.9 (85.5)
Multiplicity	3.7 (3.6)
〈*I*/σ(*I*)〉	3.70 (0.8)†
CC_1/2_	0.98 (0.34)
*R*_r.i.m._	0.272 (1.858)
*R*_p.i.m._	0.125 (0.866)
Overall *B* factor from Wilson plot (Å^2^)	60

† The resolution at which 〈*I*/σ(*I*)〉 falls below 2.0 is 3.4 Å. The cutoff value for 〈*I*/σ(*I*)〉 was based on a half data set correlation coefficient (CC_1/2_) cutoff value of 0.34.

**Table 4:** Structure solution and refinement Values in parentheses are for the outer shell.

Resolution range (Å)	47.36–3.00 (3.07–3.00)
σ Cutoff	*F* > 0.000σ(*F*)
No. of reflections, working set	31857 (2425)
No. of reflections, test set	1641 (127)
Final *R*_cryst_	0.230 (0.379)
Final *R*_free_	0.272 (0.378)
Estimated standard uncertainty	0.52
No. of non-H atoms	
Protein	10968
Ligand (Na)	6
Solvent	6
Total	10980
R.m.s. deviations	
Bonds (Å)	0.007
Angles (°)	1.5
Average *B* factors (Å^2^)	
Protein	83
Ligand (Na)	44
Solvent	26
Ramachandran plot	
Most favoured (%)	97.2
Allowed (%)	2.7
Disallowed (%)	0.1

**Table 5:** Comparison of crystallization conditions and crystallographic parameters of *Ec*DSCD

Crystallization conditions	Space group	Unit-cell angles (°)	Unit-cell dimensions (Å)	PDB code	Resolution (Å)
1 *M* sodium acetate, 50 m*M* cadmium sulfate, 50 m*M* HEPES pH 7.5	*P*3_1_21	α = β = 90, γ = 120	*a* = *b* = 112.18, *c* = 134.41	1c4t (intended)	3.0
5% PEG 4000, 0.2 *M* ammonium acetate, 0.15 *M* magnesium acetate, 50 m*M* HEPES pH 7.0	*F*432	α = β = γ = 90	*a* = *b* = *c* = 220.58	1scz (intended)	2.2
1.2 *M* ammonium sulfate, 1% ethanol, 50 m*M* potassium phosphate pH 7.0	*F*432	α = β = γ = 90	*a* = *b* = *c* = 222.80	1e2o (intended)	3.0
50 m*M* Tris pH 8.0, 2.6 *M* NaCl	*I*4	α = β = γ = 90	*a* = *b* = 128.60, *c* = 249.73	6pbr (contaminant)	3.0
