# Supplementary material for: Structure of the dihydrolipoamide succinyltransferase catalytic domain from Escherichia coli in a novel crystal form: a tale of a common protein crystallization contaminant
Source: Acta Crystallogr F Struct Biol Commun. 2019 Aug 29;75(Pt 9):616–24. doi: 10.1107/S2053230X19011488 (PMC6718150; doi:10.1107/S2053230X19011488)
Supplement: Supplementary file 1 [file f-75-00616-sup1.pdf]

Acta Cryst F

STRUCTURAL BIOLOGY COMMUNICATIONS

Volume 75 (2019)

Supporting information for article:

Structure of the dihydrolipoamide succinyltransferase catalytic domain from *Escherichia coli* in a novel crystal form: a tale of a common protein crystallization contaminant

Babak Andi, Alexei Soares, Wuxian Shi, Martin Fuchs, Sean McSweeney and Qun Liu

a

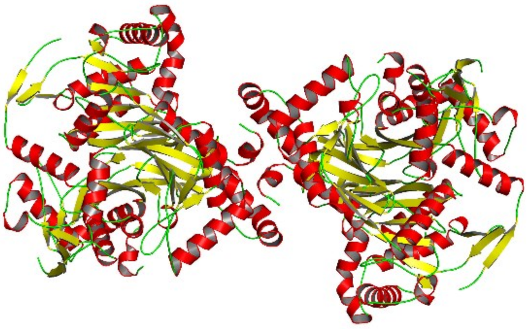

b

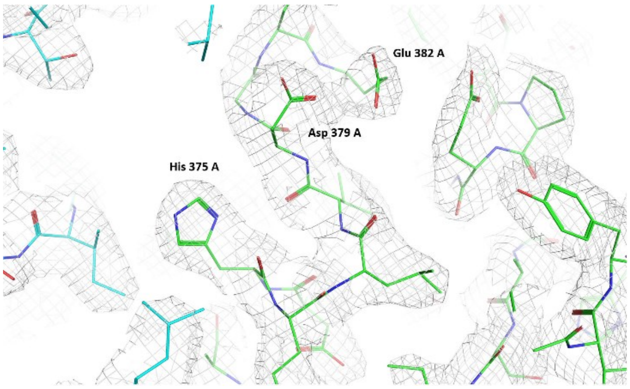

c

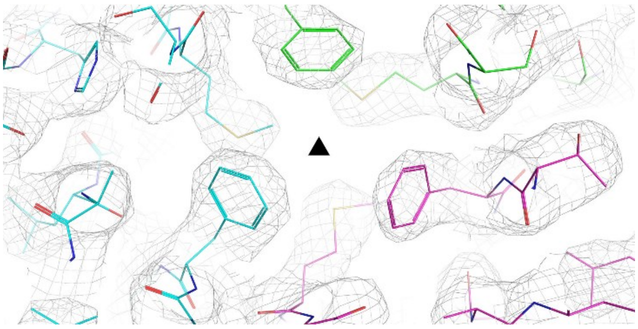

Figure S1 (a) Content of the asymmetric unit in the *I*4 crystal form which consists of six monomers. (b) A sample of the electron density map ($2F_o$ - F_c) contoured at 1 r.m.s.d.. The area shown is region 4 of the active site (Knapp *et al.*, 2000). It has been suggested that His375 is the catalytic residue. (c) A sample of the electron density map shown along the three-fold NCS axis of one trimer.
